# The bacterial communities of the small intestine and stool in children with short bowel syndrome

**DOI:** 10.1371/journal.pone.0215351

**Published:** 2019-05-16

**Authors:** Steven L. Zeichner, Emmanuel F. Mongodin, Lauren Hittle, Szu-Han Huang, Clarivet Torres

**Affiliations:** 1 Departments of Pediatrics and Microbiology, Immunology, and Cancer Biology, University of Virginia, Charlottesville, Virginia, United States of America; 2 University of Maryland School of Medicine, Institute for Genome Sciences Department of Microbiology & Immunology, Baltimore, Maryland, United States of America; 3 Department of Microbiology, Immunology, and Cancer Biology, George Washington University, District of Columbia, United States of America; 4 Gastroenterology, Hepatology and Nutrition, Children’s National Medical Center, District of Columbia, United States of America; Northwestern University Feinberg School of Medicine, UNITED STATES

## Abstract

Short bowel syndrome (SBS) presents an increasing problem in pediatrics. SBS often results from surgical resection of necrotic bowel following necrotizing enterocolitis or treatment of anatomic gastrointestinal defects. SBS is associated with significant morbidity and mortality, and creates substantial burdens for patients, families, and the health system. Recent reports have demonstrated that the fecal microbiome of children with SBS is significantly different from healthy control and severe intestinal microbial imbalances is associated with poor growth. We hypothesized that children with SBS and adverse clinical features such as PN dependent, shorter bowel length and lack of ileocecal valve would demonstrate more gut dysbiosis compare with the SBS non-PN dependent. An improved understanding of SBS pathogenesis would enhance management and potentially suggest new interventions. We studied microbial communities of SBS and control non-SBS patients from the jejunum, obtained endoscopically or by ostomy aspiration, and stool. We enrolled SBS patients who did and did not require parenteral nutrition (PN), as a surrogate marker for the seriousness of their disease. We studied the microbiota using high-throughput DNA sequencing of 16S rRNA genes and statistical analyses. We found that microbial diversity was significantly greater in jejunal aspirate than in stool samples in SBS patients, unlike non-SBS patients; that SBS patients receiving enteral feeds had greater diversity, and that SBS patients on PN and enteral feeds had lower differences in diversity in jejunal vs. stool samples. We found a trend toward increased diversity in patients with an intact ileocecal valve, and found that certain taxa were more abundant in the certain sample types, and in SBS patients vs. non-SBS patients. SBS patients have lower microbial diversity, especially patients with more severe disease, patients requiring PN, and those lacking an ileocecal valve. SBS patients, particularly those with more complex characteristics, exhibit differences in their intestinal microbiota. Particular individual taxa were over- and under-represented in patients with more unfavorable disease. While diminished diversity and alterations in microbiota composition are likely consequences of SBS, future efforts aimed at increasing microbial diversity and interventions targeting specific microbiota characteristics might constitute a testable approach to ameliorate some clinical SBS clinical consequences.

## Introduction

Short Bowel Syndrome (SBS) is characterized by a loss of intestinal length, resulting in malabsorption of nutrients, fluids and/or electrolytes. While long term outcomes have improved with the creation of multidisciplinary Intestinal Rehabilitation Programs [1–3] employing newer surgical and medical approaches [4], infants with SBS, often due to necrotizing enterocolitis or other surgical disorders of the first weeks of life, continue to experience significant morbidity and mortality [5]. Parenteral nutrition (PN), while essential for the most severely affected SBS patients, has associated adverse effects, including liver disease and infections attributed to the prolonged presence of central venous access devices [6]. The long term economic social and economic impact of PN-dependent SBS is substantial [7].

Small intestine microbiota play a major role in SBS patient outcomes. For example, small bowel bacterial overgrowth (SBBO) is a common complication associated with SBS and SBBO predicts increased morbidity and mortality in these patients. SBBO may compromise digestive and absorptive function and can delay or prevent weaning from PN [8]. SBBO may also increase rates of bacterial translocation and blood stream infections [9]. SBBO is a clinical diagnosis characterized by signs of malabsorption, including diarrhea, weight loss, abdominal pain, dehydration, bloating and other symptoms. SBBO can be identified using indirect measurements of bacterial metabolism: lactulose or hydrogen breath tests. Jejunal fluid culture aids in the diagnosis [10, 11]. Diagnosis of bacterial overgrowth is classically based upon demonstration of an increase of bacterial content by aspiration and direct culture of jejunal contents, but these methods have several limitations that include the potential contamination of the specimen by oropharyngeal bacteria during intubation, and the fact that the bacterial overgrowth may be patchy and therefore missing by a single aspiration [12]. Many consider >10^5^ CFU/ml characteristic of SBBO, but definitions vary, ranging from 10^3^ CFU/ml of specific species to 10^8^ CFU/ml [10, 11]. Cut-offs have not been validated or well-studied, and depend on the managing clinician [13], and culture methods are not standardized among microbiology labs. For these reasons, a variety of non-invasive diagnostic tests have also been suggested for the diagnosis of SBBO; these are based largely on the excretion of hydrogen in exhaled breath generated by the metabolism of carbohydrate by the luminal bacteria. The hydrogen breath test is the most common alternative method to diagnoses SBBO. It uses carbohydrate (glucose, lactulose and xylose) as a substrate [12]. In general, both the glucose and lactulose hydrogen breath tests have shown unsatisfactory abilities to predict SBBO [12, 14].

Characterization of the intestinal microbiota in SBS has been limited, mostly involving adult patients [15, 16]. Early work in an animal model of SBS found significant dysbiosis of the gut microbiota following bowel resection with decreased colonic microbial diversity [17]. Treatment of SBBO commonly involves empiric antibiotics, often without knowledge of specific offending microbes or their antimicrobial susceptibilities, and no information exists concerning optimum duration of therapy [13, 18]. In SBS patients, empiric rotating broad-spectrum antibiotics have been used in an effort to limit malabsorption and the risk of selecting multi-drug resistant pathogens [8]. Early detection and appropriate treatment of SBBO can avoid complications, including increased duration of parental nutrition and PN-associated liver damage [8, 19]. SBBO can cause villus atrophy, inflammation and mucosal damage, which increases malabsorption and the need for parenteral nutrition [20]. However, it is unknown if specific bacterial changes are correlated with increased intestinal damage and prolonged duration of parental nutrition. With such significant morbidity and mortality, many therapies aimed at ameliorating the effects of SBS and SBBO have been explored, including approaches aimed at modifying the GI microbiota of SBS patients. Small studies in some instances suggest that probiotics may have some role in treating the disorder, but substantially more work is required [21]. For example, in recent reports, no significant changes in bacterial species composition or in the proportional representation of genes encoding known enzymes were observed in the feces of humans consuming probiotics (fermented milk products) [22–24]. Detailed studies of microbial communities in SBS patients, particularly in ill patients, or comparisons of more and less ill patients, that might be useful in developing effective interventions aimed at modifying the microbial community to enhance clinical outcomes has not yet been done. In addition, most studies have focused on stool. More detailed knowledge of the microbial communities as they exist in the upper GI tract, where the losses in bowel have occurred and where most nutrient absorption takes place would clearly be important in understanding SBS. Most GI flora cannot currently be cultured; the microbial community in the GI tract has only become known with the development of culture-independent methods, like high throughput DNA sequencing of 16S rRNA genes [25]. New technologies enable identification organisms responsible for serious infection and directing focused antimicrobial therapy.

Recent reports have demonstrated that the fecal microbiome of children with SBS is significantly different from healthy control and severe intestinal microbial imbalances is associated with poor growth [26, 27]. We hypothesized that children with SBS and adverse clinical features such as PN dependence, shorter bowel length and lack of ileocecal calve, would demonstrate more gut dysbiosis compare with the SBS non-PN dependent. It is known that the intestinal microbiome change significantly during the first 2 years at which point the composition becomes relatively stable [28]. This study examines the microbiota of the small intestine, obtained by aspiration at endoscopy, and the colon in pediatric SBS patients between 2 to 10 year of age. By better characterizing the SBS microbiota, it may be possible to develop a profile of the intestinal alterations associated with more unfavorable disease. Such knowledge may lead to the development of new diagnostic tools and management strategies to decrease morbidity and mortality in these patients.

## Materials and methods

### Subjects and samples

A prospective case-cohort study was designed using metagenomics to characterized the gut microbiota of pediatric patients with short bowel syndrome and healthy controls. Twenty-nine research subjects between 2 to 10 year of age were enrolled from January 2014 to November 2015 at a single clinical trial site at Children’s National Health System (CNHS), Washington DC. The sample was a convenience sample. SBS or non-SBS patients who were about to undergo endoscopy were approached, and those who consented were enrolled. Twenty-five of the subjects had short bowel syndrome (SBS) and were recruited from the Intestinal Rehabilitation Program (IRP). Four patients had no previous surgical history and were enlisted from the general gastrointestinal (GI) service. All patients were not in the immediate post-op period, had previously been discharged to home with a stable clinical status, with all laparotomy/laparoscopy wounds completely healed, and on stable nutrition. All SBS patients received daily supplemental gastric (G)-tube or jejunal (J)-tube feeds of elemental amino acid base formulas; nine of them received daily PN (see Table 1 for details). All patients older than 6 months have an oral diet consistent in cow’s milk-free diet, sugar-free diet, no juices, no raw vegetables and drink oral rehydration solution (ORS), with specifications established by the World Health Organization [29].

**Table 1:** Patient demographics.

	Enteral Nutrition (n = 16)	Parenteral Nutrition (n = 9)	Healthy Controls (n = 4)
Mean Age (years)	5.75	3.8	6.75
Male Gender	9 (56%)	9 (100%)	1 (25%)
Race/Ethnicity			
African American	9 (56%)	5 (56%)	2 (50%)
Caucasian	5 (31%)	3 (33%)	2 (50%)
Hispanic/Latino	3 (19%)	1 (11%)	0
Other	2 (13%)	1 (11%)	0
No Acid Blocker	9 (56%)	2 (22%)	1 (25%)
Bacterial Overgrowth Prophylaxis	16 (100%)	9 (100%)	0 (0%)
Probiotic Prophylaxis	13 (81%)	4 (44%)	1 (25%)
BMI Range	16–26.4	15.3–20.5	14.2–22.9

Demographic information is summarized for all patients with samples yielding microbial DNA that was subject to 16S rRNA gene sequencing.

Jejunal specimens were collected from all patients either by upper endoscopy procedure (if they require an endoscopy as part of the current standard of care) or immediately after a placement of a new G/J tube. Twenty-six of the 29 patients provided paired jejunal and stool specimens. The jejunal specimens were frozen kept at -80C pending DNA extraction. All recruitment and study procedures were approved by the Children’s National Health System/Children’s National Medical Center Institutional Review Board, and the informed consents were obtained from parents/guardians.

Study entry criteria included: diagnosis of short bowel syndrome resulting from surgical resection of NEC or a congenital anomaly such as gastroschisis or intestinal atresia, ages 2–10 years, stable at home for more than 4 weeks with no signs of active infections (sepsis, central line associated blood stream infection (CLABSI), acute viral or bacterial infection that can cause hospitalization). Nine patiets of this cohort received on a daily basis PN and enteral feeds (PO and G tube feeding) and 16 SBS patients received only oral enteral feedings and/or G tube feedings at the time of obtaining the sample. We use a requirement for PN as a marker for SBS patients who were more severely affected by their disease. Samples were obtained at least 2 weeks following the cessation of antibiotic treatment for SBBO prophylaxis. Control patients were obtained from the GI service, who had a medical indication for endoscopy, since performing an invasive procedure on a child for research purposes only, without therapeutic intent, would not be ethical. These patients were between 2–10 years of age, were stable at home and had no history of recent or active infections for at least 1 month. These patients had an upper endoscopy as part of the standard-of-care evaluation for hematochezia, abdominal pain, dysphagia or poor weight gain, but had normal visual endoscopic findings and normal intestinal histology. Study exclusion criteria included recent hospital admission (within 1 month) due to bacterial or viral infections, including CLABSI, enterocolitis, infectious diarrhea; and intestinal failure patients, PN-dependent, with no history of bowel resection. Exclusion criteria for healthy controls included use of antimicrobial agents, or steroids (oral, nasal, or inhaled) within 1 month of study.

Jejunal and stool samples were taken to the lab and frozen for later bacterial DNA extraction and sequencing. Fresh jejunal specimens were also sent to the CNHS laboratory for bacteriological cultures (colony count culture and antimicrobial agent sensitivity).

Demographic and clinical data (age, gender, ethnicity, diet (enteral vs. parenteral), antibiotic history, surgical history, concomitant medications, co-existing medical conditions, results from the traditional jejunal cultures and length of the remaining small bowel/ colon) for each patient was abstracted from the medical record by a single reviewer.

### DNA extraction and 16S rRNA gene amplification

Total genomic DNA was extracted at CHNS using a protocol developed at the University of Maryland School of Medicine—Institute for Genome Sciences and previously described [30]. Briefly, samples were thawed on ice, incubated in an enzymatic cocktail containing lysozyme, mutanolysin, proteinase K and lysostaphin, after which the microbial cells were lysed using bead beating with silica beads (Lysing Matrix B, MP Biomedicals) with the FastPrep instrument (MBio, Santa Ana, CA). The DNA was then further extracted and purified using the Zymo Fecal DNA kit (Zymogen).

### 16S rRNA gene PCR amplification and sequencing

Microbiota profiling was performed by PCR amplification of the V3V4 hypervariable region of the 16S rRNA gene, followed by sequencing on Illumina HiSeq 2500 (San Diego, CA, USA) modified to generate 300 bp paired-end reads [31]. PCR amplification of the V3V4 region of the 16S rRNA gene was performed using a two-step PCR reaction in which sample barcoding is performed during the second PCR to maximize target amplification [32]. Briefly, the first PCR was performed using modified 16S rRNA gene specific primers (319 F: (ACACTGACGACATGGTTCTACA[0–7]**ACTCCTRCGGGAGGCAGCAG** and 806 R: TACGGTAGCAGAGACTTGGTCT[0–7]**GGACTACHVGGGTWTCTAAT**) where the underlined sequence is the Illumina sequencing primer sequence and [0–7] indicate the heterogeneity spacer sequence aimed at minimizing biases associated with Illumina sequencing of low-diversity amplicons [33]. The first-step PCR was performed using the Phusion high-fidelity PCR master mix (Thermo Fisher, USA) and 9 ul of extracted DNA as template in a total reaction volume of 25 ul, using the following cycling parameters: 3 min at 95°C, followed by 20 cycles of 30s at 95°C, 30s at 58°C, and 1 min at 72°C, with a final step of 5 min at 72°C. This was followed by a low-cycle second PCR using primers targeting the Illumina sequencing primer sequence from the first step amplicon (H1: AATGATACGGCGACCACCGAGATCTACACNNNNNNNNACACTGACGACATGGTTCTACA and H2: CAAGCAGAAGACGGCATACGAGATNNNNNNNNTACGGTAGCAGAGACTTGGTCT) where NNNNNN indicates a sample specific barcode sequence. A 1:20 dilution of the step one PCR products was performed prior to step two amplification. Second-step PCRs were set up using the Phusion high-fidelity PCR master mix (Thermo Fisher, USA) and 1 ul of diluted first-step amplicon product. Cycling parameters for the second-step PCR were: 30 s at 95°C, followed by 10 cycles of 30 s at 95°C, 30 s at 58°C, and 1 min at 72°C, with a final step of 5 min at 72°C. No-template negative controls were included for each PCR and each primer pair. The presence of PCR amplicons was confirmed using gel electrophoresis, after which the SequalPrep normalization plate kit (Life Technologies, Inc.) was used for cleanup and normalization (25 ng of 16S PCR amplicon pooled for each sample) before sequencing.

### Data processing and statistical analysis

Following sequencing, 16S rRNA reads were processed and analyzed using minor modifications of previously published methods [34–36]. Briefly, sequencing reads were initially screened for low-quality bases and short read lengths [33], after which paired-end read pairs were assembled using PANDAseq [37]. The resulting consensus sequences were then demultiplexed, trimmed of barcodes and primers, and assessed for chimeras using UCHIME [38] in de novo mode implemented in QIIME (v. 1.9.1) [39]. Quality-trimmed sequences were then clustered de novo into operational taxonomic units at 97% similarity cutoff using USEARCH [40] implemented in QIIME, and taxonomic assignments were performed using the RDP classifier implemented in QIIME and the Greengenes database (v. 13.8) database as a reference. The resulting taxonomic assignments were imported as a BIOM-formatted file into R (v. 3.5.0) using RStudio (v. 1.1.456) integrated development environment (IDE), and processed/analyzed using the following R packages: Phyloseq (v. 1.24.2), Vegan (v. 2.5–2), and gpplot2 (v. 2.2.1). When appropriate, taxonomic assignment data were normalized to account for uneven sampling depth with metagenomeSeq’s cumulative sum scaling (CSS; implemented in R) [41], a normalization method that has been shown to be less biased than the standard approach (total sum normalization). Good’s coverage index was calculated for each sample in order to ensure appropriate sequence coverage: samples with Good’s coverage <0.95 were discarded from the analyses. In addition, ultralow abundant and likely to be spurious OTUs (<0.005% relative abundance and present in <10% of samples) were removed from the OTU table prior to the analyses described below.

Alpha-diversity (within-sample comparisons) analyses were performed on non CSS-normalized datasets using the Observed and Shannon diversity indices calculated using Phyloseq. Beta-diversity (between-sample) comparisons were performed from CSS-normalized data through principal-component analysis (PCoA) plots of Bray-Curtis, Unweighted and Weighted UniFrac distances determined using QIIME and tested for significance using the ANOSIM algorithm (9,999 permutations) implemented in the Vegan package in R. Determination of statistically significant differences for OTU bacterial relative abundance levels was performed using DESeq2 [42] implemented in R using an adjusted p value <0.05.

For pairwise tests of statistical significance for difference sample types and clinical conditions we determined p-values using the Kruskal-Wallis rank sum test and plotting and linear modeling conducted using R and its packages, including tidyverse with ggplot2 and its included geom_boxplox, and stats.

To compare the slopes of the short bowel length vs. diversity a linear regression model was used: 'Value' = b0 + b1* OnPN + b2* short_bowel_length + b3*OnPN x short_bowel_length, where 'OnPN' is an indicator variable, equal to 1 if the sample is from a patient on PN and 0 otherwise. The interaction term is the multiplication of the indicator variable with the short_bowel_length variable. P-values for linear modeling were calculated and compared using SAS.

### Accession number(s)

Sequence data generated in this study were deposited with GenBank and linked to BioProject number BioProject ID: PRJNA492751 in the NCBI BioProject database.

## Results

Following DNA isolation, 16S rRNA gene amplification and sequencing, the 55 samples yielded 2,919,920 non-chimeric sequences clustered in a total of 5321 operational taxonomic units (OTUs) using a cutoff sequence identity of 97% (roughly equivalent to species-level OTU clustering). The average number of sequences per specimen was 54,072.

Of the 25 SBS patients, 16 were not receiving parental nutrition (PN), and 9 were receiving partial parental nutrition with some enteral feedings (oral or supplemental gastric (G)- or jejunal (J)-tube feeds). Table 1 lists patient characteristics. In general, patients receiving parental nutrition tended to be younger than patients on entirely enteral feeds and the healthy controls. In accordance with standard of care at CNMC, all SBS patients received antimicrobial prophylaxis for bacterial overgrowth consisting of a rotating schedule of oral antibiotics and nystatin. Many patients also received erythromycin to enhance gastric motility. However, samples were obtained at least 2 weeks following the cessation of antibiotic treatment for SBBO prophylaxis and for motility. Among non-SBS controls, one subject had been treated for *Clostridium difficile* colitis in the month prior to enrollment. The four non-SBS controls underwent endoscopy as part of the standard care evaluation for hematochezia, abdominal pain, dysphagia or poor weight gain. Pathology from biopsy samples identified a benign polyp in the subject with hematochezia, but was otherwise normal. The subject with poor weight gain had a history of a restrictive diet with periods of good weight gain in the past, but underwent endoscopy to rule out a biological process prior to referral for nutritional counseling.

Table 2 lists the clinical characteristics of the SBS patients. Patients who had fully transitioned to enteral feeds had a mean jejunal length of 67.2 cm, compared to patients who continued to receive partial parental nutrition, whose mean jejunal length was 50.1 cm. Patients had received an average of 27.5 months of parental nutrition prior to successful transition to full enteral nutrition. Although duration of parental nutrition is provided for patients on parental nutrition, it should be noted that these patients continued to receive therapy at the time of sampling, and the mean duration of 39.6 months reflects ongoing therapy.

**Table 2:** Patient characteristics.

	Enteral Nutrition (n = 16)	Parental Nutrition (n = 9)
Underlying Diagnosis		
Necrotizing Enterocolitis	5 (31%)	2 (22%)
Gastroschisis	2 (13%)	3 (33%)
Intestinal Atresia	5 (31%)	3 (33%)
Volvulus	3 (19%)	1 (11%)
Hirschsprung’s disease	1 (6%)	0
Other	0	1 (11%)
Mean Jejunal Length (cm)	67.2	38
Ileocecal Valve		
Yes	6 (38%)	2 (22%)
No	4 (25%)	4 (44%)
Unknown	6 (38%)	3 (33%)
Average Duration of Parental Nutrition (months)	27.5	39.6

The table presents summary data for key characteristics of the patients enrolled in the study, including the underlying diagnosis, mean estimated jejunal length, the presence or absence of an ileocecal valve, and the duration of parenteral nutrition.

**Fig 1:**
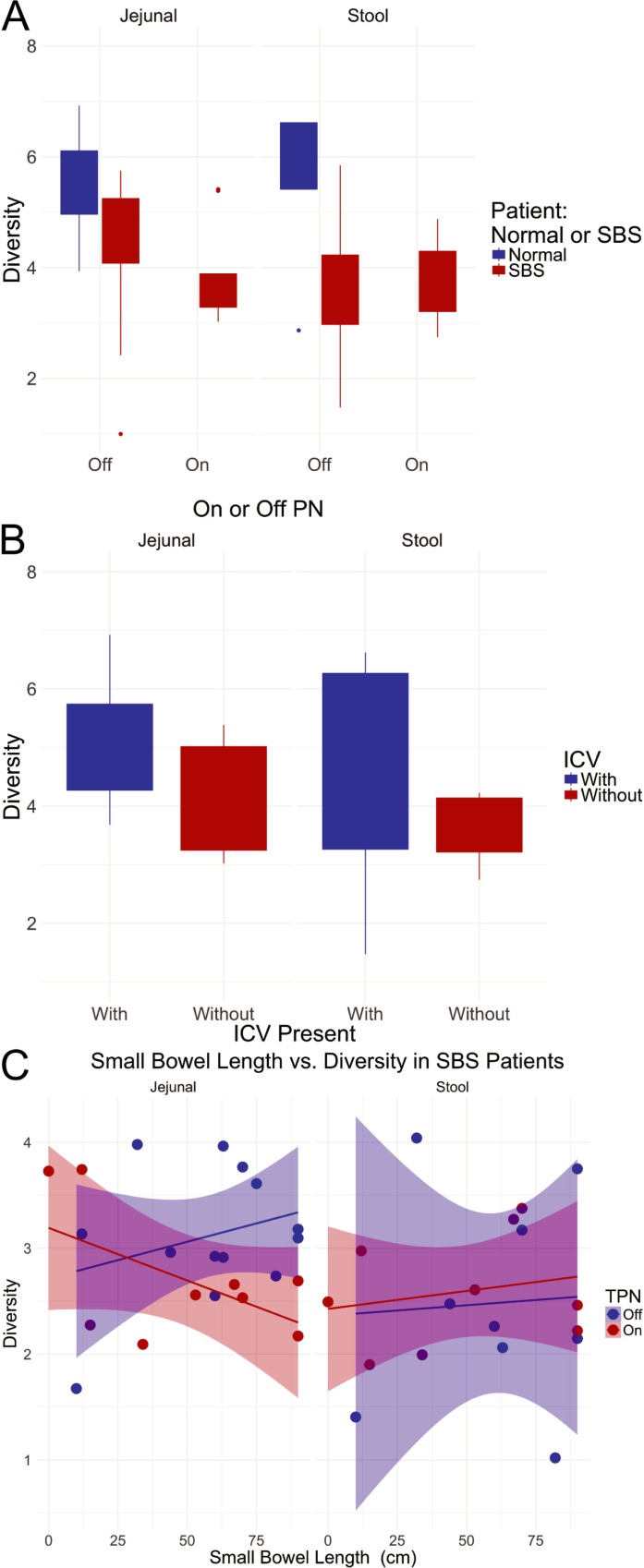
A. The figure shows box and whisker plots (upper and lower hinges show 25^th^%tile and 75^th^%tile, whiskers extend 1.5*IQR from the hinge, outlier values plotted individually) for the Shannon diversity scores (“Diversity” on the y-axis) of the indicated sample types and patient characteristics. B. The figure shows box and whisker plots (upper and lower hinges show 25^th^%tile and 75^th^%tile, whiskers extend 1.5*IQR from the hinge, outlier values plotted individually) for the Shannon diversity scores (“Diversity” on the y-axis) of the indicated sample types and patient characteristics. C. The figure shows a plot of microbial diversity (Shannon) vs. small bowel length for JA and SS for patients requiring PN or not. Linear regression lines are plotted for each sample type, with the shaded areas showing 95% confidence intervals.

Ecological diversity (Shannon and Simpson scores) and richness (Chao1 score) indices were calculated for each sample; median values and standard deviations are provided by nutritional status in Table 3. Both Simpson and Shannon diversity indices provide insights into species composition and relative abundance of bacterial communities. The Shannon index emphasizes rare species by slightly reducing the "weight" of abundant species relative to more rare species. The Simpson index emphasizes the opposite; the weight of rare species is reduced relatively more than that of more abundant species [43]. The Chao1 estimator describes the community richness, e.g. the total number of species present in a sample. Table 3 provides the range of diversity scores for patient populations and sampling sites. Mean Chao1 scores ranged from 498.87 to 926.3, with healthy controls having higher average Chao1 scores. Mean Shannon diversity scores ranged from 3.61 to 5.6 and mean Simpson scores ranged from 0.78 to 0.93. The Shannon and Simpson scores in the healthy controls also tended to be greater than SBS patients, although a difference between SBS patients receiving parental versus enteral nutrition was not apparent. Stool samples (SS) tended to have lower scores than jejunal aspirate (JA) samples, however the difference was mostly observed in patients receiving enteral nutrition; healthy controls and parental nutrition patients demonstrated higher scores in SS (Fig 1A).

**Table 3:** Diversity and Richness Estimators.

	Average Number of OTUs	Shannon	Chao1	Simpson
Parental Nutrition				
All		3.83 (2.74–5.41)	604.25 (358.11–1163.97)	0.82 (0.73–0.94)
Jejunal	145–600	3.92 (3.02–5.41)	669.31 (358.11–1163.97)	0.82 (0.73–0.94)
Stool	130–357	3.74 (2.74–4.87)	539.2 (388.1–672.74)	0.81 (0.73–0.92)
Enteral Nutrition				
All		4.02 (1.0–5.85)	569.8 (187–1080.62)	0.81 (0.25–0.96)
Jejunal	42–620	4.35 (1.0–5.75)	627.43 (187–1080.62)	0.84 (0.25–0.96)
Stool	71–568	3.61 (1.47–5.85)	498.87 (235.12–981.92)	0.92 (0.78–0.97)
Healthy Controls				
All		5.55 (2.87–6.92)	914.4 (312.22–1651.23)	0.93 (0.78–0.98)
Jejunal	202–808	5.5 (3.94–6.92)	926.3 (434.21–1651.23)	0.93 (0.86–0.98)
Stool	123–877	5.6 (2.87–6.62)	902.5 (312.2–1257.1)	0.92 (0.78–0.97)
All Jejunal Samples (n = 29)	42–808	4.38 (1.0–6.92)	681.65 (187.0–1651.23)	0.85 (0.25–0.98)
All Stool Samples (n = 26)	71–877	3.96 (1.47–6.62)	574.93 (235.1–1257.1)	0.81 (0.36–0.97)

The table shows the mean values of the Chao1, Shannon and Simpson diversity for the specified samples.

The Kruskal-Wallis rank sum test was used to test for statistical significance between patient subpopulations (Table 4), which were chosen for comparison based on clinical importance. Significant differences were appreciated between jejunal samples of the healthy controls and SBS patients receiving parental nutrition. No differences were appreciated in SS, or between JA and SS. There was a trend towards significance when comparing jejunal to stool specimens in patients receiving enteral feeds, but there were not enough healthy controls enrolled to further examine the relationship between jejunal and stool microbiomes.

**Table 4:** Shannon Diversity Scores.

	p-value
Jejunal Samples:	
Parental vs. Enteral Nutrition	0.10
Parental Nutrition vs. Healthy Controls	**0.03**
Enteral Nutrition vs. Healthy Controls	0.16
All Samples	**0.052**
Stool Samples:	
Parental vs Enteral Nutrition	0.76
Parental Nutrition vs. Healthy Controls	0.12
Enteral Nutrition vs. Healthy Controls	0.07
All Samples	0.17
Jejunal vs. Stool	
All Samples	0.46
Parental	0.57
Enteral	0.08
Healthy Controls	N/A
SBS Patients With and Without Ileocecal Valves	
Jejunal Samples	0.13
Stool Samples	0.37

The table compares the Shannon diversity scores for selected, clinically interesting patient and sample types. The Kruskal-Wallis test was used to calculate p-values.

We also compared diversity in SBS patients with and without ileocecal valves, since this has been observed to be an important difference in clinical behavior of SBS patients. While a possible trend was apparent (Fig 1B), the differences did not achieve statistical significance for either the jejunal (p-value = 0.1325) or the stool (p-value = 0.3718) samples.

The functional characteristics of the microbial community of the GI tract in SBS may be reasonably thought to depend upon the amount of bowel remaining after resection. We therefore examined the Shannon diversity scores as a function of the remaining small bowel length for the SBS patients (Fig 1C). For this analysis we omitted one apparent outlier value with a small bowel length estimated at 190 cm, more that twice the value of the next longest small bowel. We found that for SS there was no trend to increased diversity associated with length (slope estimate = 0.0037, p-value = 0.649 for slope ≠ 0 for patients not requiring PN and slope estimate = 0.0049, p-value = 0.692 for slope ≠ 0 for patients requiring PN). For the jejunal samples we observed an apparent difference in the association of microbial diversity and small bowel length. For patients not requiring PN, we observed a modest trend to increased diversity with increasing small bowel length, but for patients requiring PN we observed a somewhat counter-intuitive trend, with increased small bowel lengths associated with decreasing diversity (slope estimate = 0.0101, SE = 0.0009, p-value = 0.296 for slope ≠ 0 for patients not requiring PN and slope estimate = -0.0144, SE = 0.0095, p-value = 0.129 for slope ≠ 0 for patients requiring TPN), with the difference in slopes (On PN–Off PN = -0.0246, SE = 0.0131, p = 0.077).

**Fig 2:**
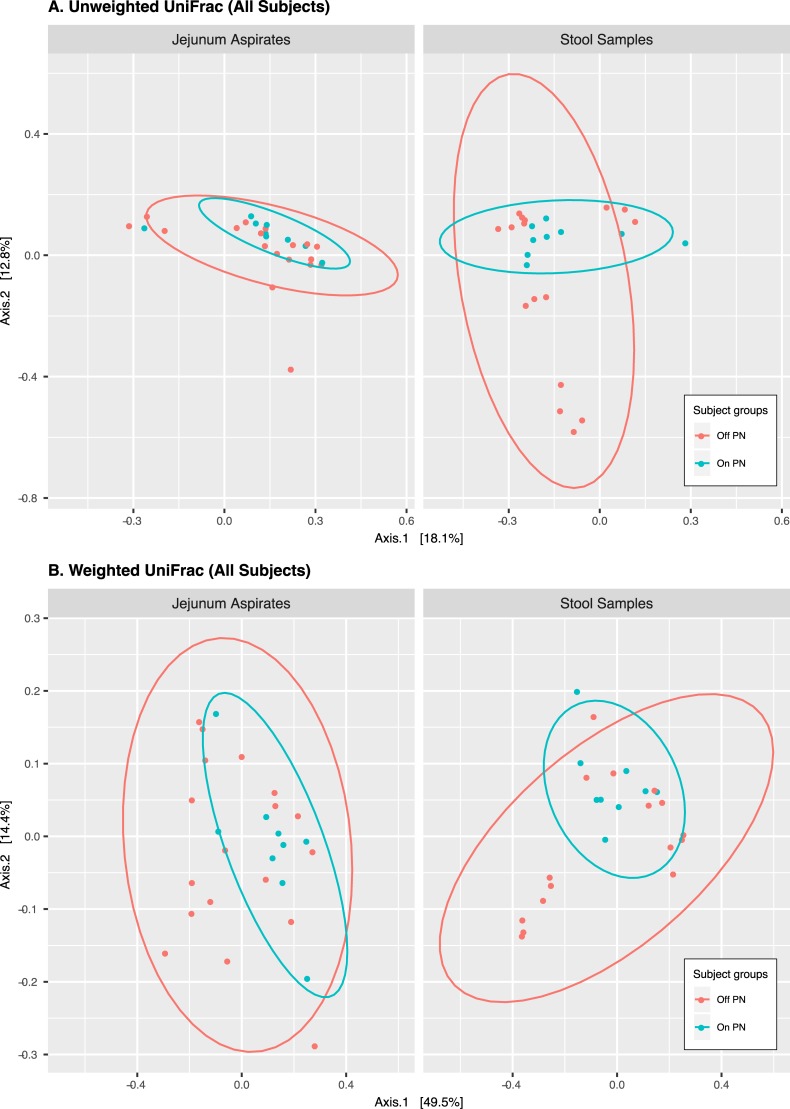
The figure shows box and whisker plots (upper and lower hinges show 25^th^%tile and 75^th^%tile, whiskers extend 1.5*IQR from the hinge, outlier values plotted individually) for the Shannon diversity scores (“Diversity” on the y-axis) of the indicated sample types and patient characteristics.

One important question concerning the microbial communities in SBS is whether distinctive microbial communities are associated with particular disease characteristics. We conducted several analyses aimed at identifying features of microbial communities that may be characteristic of SBS-related disease states. We examined the data using data reduction approaches, including principal component analyses/dimensional reduction (multidimensional scaling principal component analysis (MDS/PCoA) and t-Distributed Stochastic Neighbor Embedding (t-SNE)), using Jensen-Shannon Divergence, Bray-Curtis, and Unweighted and Weighted UniFrac metrics, comparing the communities in the present in jejunal aspirates (JA) and the stool samples (SS), for all patients, for patients receiving or not receiving PN, and for SBS patients alone. Fig 2 shows the results for the Unweighted and Weighted UniFrac scores. Dimensional reduction approaches, we observed some trends toward distinctive community characteristics, suggests that the communities of patients receiving PN tend to be more similar to each other, but overall there did not appear to be distinctive microbial community structures that characterized the SBS communities.

**Fig 3:**
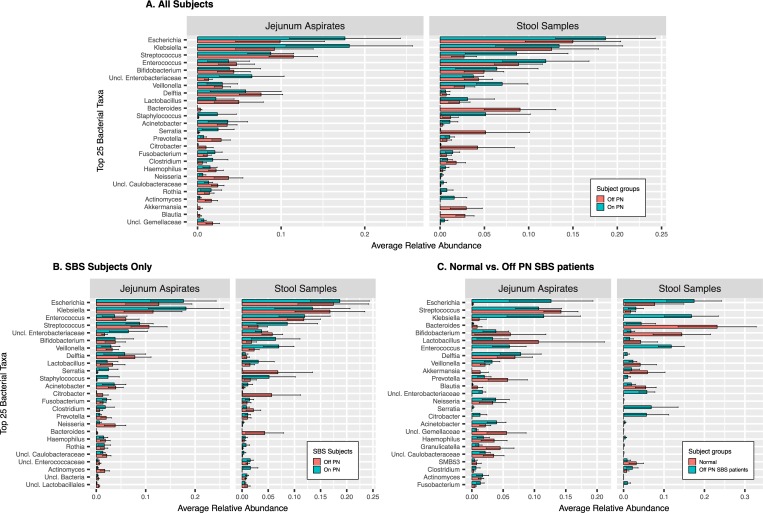
Average Relative Abundance of the Top 25 Most Abundant Taxa in SBS Patients in All Samples and Sites and in SBS Patients with Different Clinical Characteristics. A. Relative abundance of the top 25 bacterial taxa in JA and SS in all patients, comparing patients receiving and not receiving PN. B. Relative abundance of the top 25 bacterial taxa in JA and SS in SBS patients only, comparing patients receiving and not receiving PN. C. Relative abundance of the top 25 bacterial taxa in JA and SS in all patients, comparing SBS patients not receiving PN and normal patients.

Since identifying particular individual taxa associated with SBS, or with better or worse SBS-related clinical manifestations, might inform future clinical interventions we examined the data for taxa that might be over- or under-represented in different disease states. To provide an overview of some of the differences in the taxa in the different patient types, we examined and plotted the 25 most abundant taxa in all the samples (Fig 3A), for the JA and SS, comparing patients who were receiving parenteral nutrition (On PN) with those who were not (Off PN). No detailed statistical analysis was conducted on these top 25 taxa. All identified taxa were studied in the DeSeq2 differential abundance analysis presented below. Although there was substantial patient-to-patient variability, we found that, for the JA samples, certain unclassified Enterobacteriaceae (that is, a particular individual taxa identified as a member of the Enterobacteriaceae, but not further classified at the Genus level or more at this time), Serratia, and Staphylococcus had a higher abundance in patients receiving PN and Neisseria, Actinomyces, Akkermansia, and certain unclassified Gemellaceae had a higher abundance in patients not receiving PN. For the SS samples, we found that Veillonella, Staphylococcus, Rothia, and Actinomyces had higher abundance in patients receiving PN, and Bacteroides, Citrobacter, Akkermansia, and Blautia had a higher relative abundance in patients not receiving PN. Considering SBS patients only (Fig 3B) we found in JA samples higher relative abundances of an unclassified Bacteriaceae, Serratia, and Staphylococcus in patients receiving PN, and Neisseria and Actinomyces in patients not receiving PN. In the SBS patient SS, we found that there were higher relative abundances of Bifidobacterium, Staphylococcus, Veillonella, and Actinomyces in patients receiving PN, and higher abundances of Citrobacter and Bacteroides in patients not receiving PN. We also examined the differences in the top 25 most abundant taxa in the JA and SS specimens comparing normal and SBS patients not receiving PN to see if, even in SBS patients with the least severe disease there were particular taxa characteristic of the SBS disease state. We found that, compared to the normal patients, in JA samples Klebsiella, Enterococcus, an unclassified Enterobacteriaceae, and Citrobacter were present in higher abundance in the SBS patients and Lactobacillus, Prevotella, an unclassified Gemellaceae, and Granulicatella were present in higher abundance in the normal patients. In the SS samples, Klebsiella, Enterococcus, Serratia, Citrobacter, and Fusobacterium, were present in higher abundance in the SBS patients, and Prevotella, an unclassified Gemellaceae, Akkermansia, and Granulicatella were higher in the normal patients. In SS, Klebsiella, Enterococcus, unclassified Enterobacteriaceae, Serratia, Citrobacter, and Fusobacterium were higher in the SBS patients, and Bacteroides, Bifidobacterium, Akkermansia, and SMB53 were higher in the normal patients.

**Fig 4:**
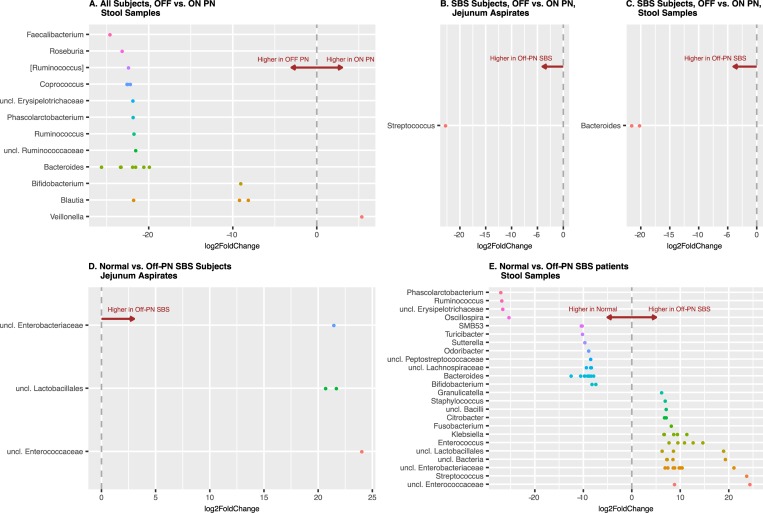
Differential taxa abundances. The plots shows the log2-fold changes for genus-level bacterial OTUs that were statistically significant at the 0.05 level using the Wald test with Benjamini-Hochberg adjustment as implemented in DESeq2. Each data point represent a genus-level OTU (y-axis) identified as significantly different along with the log2 fold change (X-axis). Negative values indicate the log2 fold change for taxa under represented in patients receiving PN. Positive values indicate the log2 fold change for taxa over represented in patients receiving PN. A. Differential abundances for all patients, comparing taxa in SS for patients on or off PN. B. Differential abundances for SBS patients only, on or off PN. C. Differential taxa abundance for SBS off PN vs. normal patients. D. Differential abundances for JA for normal patients vs. SBS patients not receiving PN. E. Differential abundances for SS for normal patients vs. SBS patients not receiving PN. The corresponding DESeq2 results table, with representative sequences for each OTU identified as significantly differentially abundant, are displayed in S1 Table.

We then examined the data for bacterial taxa, defined at the Genus level, that were significantly different in the different clinical states, per the Wald test with Benjamini-Hochberg adjustment for false discovery, as implemented in DESeq2 [42] (Fig 4, data also presented in tabular form in S1 Table). We found no significant differences when we compared JA samples from all patients on or off PN (not shown). When we examined the SS from all patients, comparing the patients on or off PN, we found taxa from several genera under represented in the patients on PN, Faecalibacterium, Roseburia, Ruminococcus, Coprococcus, Erysipelotrichaceae, Phascolarctobacterium, Ruminococcus and other Ruminococcaceae), Bacteroides, Bifidobacterium, Blautia, were under represented in the sample from the patients on PN and Veillonella was over represented in the patients on PN (Fig 4A). When we specifically compared the relative abundances of taxa at the Genus level for the JA and SS samples just for SBS patients, we found that in the JA samples Streptococcus was under represented and in the SS samples (Fig 4B). Bacteroides was under represented (Fig 4C).

We then studied whether there were taxa at the Genera level that were differentially present in the SBS patients with even the best clinical outcomes, that is SBS patients who did not require PN, or taxa that were characteristic of the state of having a short bowel. We found that in the JA samples (Fig 4D) an unclassified Enterobacteriaceae, two unclassified Lactobacillales, and an unclassified Enterococcaceae were significantly overabundant in the SBS patients, and in the SS samples (Fig 4E) we found that Phasocolarctobacterium, Ruminococcus, an unclassified Erysipelotrichaceae, Oscillospira, SMB53, Turicibacter, Sutterella, Odoribacteria, two unclassified Peptostreptococcaceae, an unclassified Lachnospiraceae, 5 Bacteroides, and 2 Bifidobacterium were overabundant in the SBS patients; and that Granulicatella, Staphylococcus, an unclassified Bacillus, Citrobacter, Fusobacterium, 4 Klebsiella, 5 Enterococcus, 3 unclassified Lactobacillales, 3 unclassified Bacteria, 4 unclassified Enterobacteriaceae, Streptococcus, and 2 unclassified Enterococcaceae were overabundant in the normal SS samples.

Finally, we examined the relationship between 16S sequencing and traditional bacterial cultures. As expected, bacterial culture was dominated by Gram-negative organisms, such as *E*. *coli* and *Klebsiella pneumoniae* and Gram-positive *Streptococcus* and *Enterococcus* species. Inspection of the phyla identified from the OTU clustering demonstrating the Firmicutes and Proteobacteria dominated the jejunal samples. The Gram-negative organisms identified on culture were members of the Proteobacteria phylum and the Gram-positives from the Firmicutes phylum. Other phylum represented in the sequencing data included Bacteroidetes, which includes anaerobic bacterial genuses which would be expected to grow poorly, if at all, on traditional aerobic bacterial cultures. Analysis of traditional bacterial culture data was restricted by limited bacterial speciation of polymicrobial cultures (S1 Table). Additionally, 16S sequencing does not allow for quantitation of bacterial load, as 16S genes may be present in variable quantities in different bacterial species. OTU clustering cannot reliably identify all sequences to the species level, therefore, it is not possible to determine the number of 16S genes present per OTU to attempt quantitation at present time.

## Discussion

While our study is limited by small sample size, features of the small bowel microbiota identified in this study are consistent with the findings of previous studies of healthy controls in adults and children, although due to difficulties in obtaining samples, relatively less is known about the small bowel microbiota compared to fecal microbiota. Historically, the small intestines including the jejunum were described as sterile [44] or absent of bacteria typically present in the lower gastrointestinal tract [45]. However, as molecular methods are applied to the microbiota of the small intestines a much more complex ecological picture is emerging.

We found that the small intestine microbiota showed a trend towards greater diversity than the colon microbiome, across a number of diversity score metrics and patient populations. Other studies of the small bowel microbiome have identified similar findings in healthy individuals. A study of healthy adult volunteers found that duodenal samples were more diverse than colonic samples, with mucosal-biopsy samples found to be more conserved than luminal samples, while luminal samples demonstrated more variability within individuals [46]. As mucosal-associated bacteria may have evolved mechanisms to optimize their growth at host mucosal boundaries, it is not surprising that luminal bacterial are more variable. A study of the duodenal microbiome of obese and lean controls demonstrated between 317 and 591 total observed OTUs, Chao scores of 365 to 655 and Shannon diversity scores of 4.0 to 4.41, which are within the range observed in this study [47]. A 2013 study of pediatric duodenal samples found that the Shannon diversity scores of their normal controls ranged from greater than 4.5 to less than 3.5 [48]. A study of pediatric intestinal transplant subjects, many of whom were receiving prophylactic antibiotics, found Shannon diversity scores ranging from greater than 2 to less than 7, and Simpson diversity scores ranging from 0.4 to less than 1.0 [49].

In our study, increasing small bowel length was associated with a slight trend toward increased diversity in SS from SBS patients that both did and did not require PN. For jejunal samples, we found that increasing small bowel length was associated with a modest trend toward increase in diversity for patients not requiring PN, and a modest trend toward a decrease in diversity for patients requiring PN. While more work will be required to fully examine this finding, with the enrollment of more subjects to potentially achieve significance, one possible hypothesis would be that for patients requiring PN, who have more severe disease, with lower small bowel lengths there could be less separation between the lower intestinal and small bowel microbial communities, resulting in more taxa characteristic of the lower intestine present in the small bowel microbial community and so increasing its diversity.

Using QIIME, after spurious OTU filtering, we were able to identify 3739 different OTUs from the available sequence data. Of those 3739 OTUs, 3627 could be identified to a phylum level, 2051 could be identified to the genus level and 569 could be identified to the species level. Bacterial phyla consist of many hundreds of genus of bacteria, each of which in turn contain multiple species of bacteria [50]. Microbiology has historically sought to link species of bacteria to specific disease manifestations. However, bacterial sequencing data studies raise questions regarding the validity of that perspective. Given the complexity of the small intestinal microbiota and the possible temporal variations with diet and other environmental factors, efforts to find differences in a single phyla, genera or species may be flawed and oversimply the richness of the small intestinal microbiome. Many studies have also demonstrated significant inter-individual variations in phyla level data [47, 51], while others have demonstrated that the small intestine microbiome itself fluctuates significantly with time and diet [51, 52]. Studies of microbiome enterotypes appears more promising, but larger sample sizes are necessary, and studies may need to examine factors such as diet and time of sampling.

In this study, analysis was complicated by clinically required pre-treatment with antibiotics, probiotics and differences in diet between the subjects and patient subpopulations. Previous studies demonstrated that diet changes can be reflected in the fecal microbiome [53]. Two studies suggested that the temporal shifts in the small intestine microbiome are even greater and more variable that those of the stool microbiome [51, 52], although both studies used microarrays rather than sequencing.

Interestingly, we found that patients receiving PN had less diverse jejunal microbiota than those receiving only enteral nutrition. It is not possible to determine whether lower small bowel diversity led to worsened clinical status, yielding a requirement for PN, or whether PN somehow decreased jejunal aspirate microbial diversity. The observation, however, suggests the intriguing hypothesis that efforts to increase small bowel microbial diversity, whether by supplying prebiotics, probiotics, or carefully designed microbial inoculations might modulate the clinical requirement for PN. We found no significant differences in small bowel diversity between SBS subjects on enteral nutrition and the healthy controls, suggesting that the pediatric small intestine has the ability to adapt and return to a more typically microbiome, as assessed by diversity metrics, when parental nutrition has been replaced with enteral nutrition.

Our study was limited, but it reflects the likely ‘real-world’ microbiota of SBS patients, who are certain to have multiple confounding factors. Further, the pediatric small intestinal microbiome should demonstrate normal developmental changes as the pediatric diet transitions to a more adult diet. There may also be a bias against rare taxa, and various technical factors may complicate data collection [54, 55]. In addition, DNA-based sequencing of the 16S rRNA gene does not differentiate between the presence and absence of living bacteria, and, as a result, the presence of bacterial genomic content may not totally reflect living and metabolically-active bacteria. Finally, another limitation of this study was the inability to perform taxonomic assignments to the level of bacterial species or even genus (only 15.2% of OTUs were assigned to species level, and 54.8% to genus level), including for OTUs identified as significantly differentially abundant between SBS paptients and controls. This is a known limitation and an unresolved challenge for 16S rRNA gene sequence taxonomic assignments [56]. While taxonomic assignments of bacterial OTUs significantly associated with SBS might need to be refined to provide a more detailed characterization of the role of these taxa in the pathophysiology of SBS, their use as a non-invasize biomaerker might nonetheless provide a useful tool for SBS, as it has been the case in other human conditions and diseases [57–60]. Future studies are necessary to better characterize the multiple factors that could influence the small intestinal microbiome, and more importantly determine patient outcomes linked to the presence of different bacterial microbiota compositions.

Nevertheless, while more studies with larger numbers of subjects are clearly needed, our findings, including those that indicate that diversity scores differ depending on GI pathology, with SBS patients having lower microbial diversity scores, that diversity scores also differ depending on the severity of disease associated with SBS (patients not requiring PN vs. patients requiring PN, patients with and without ileocecal valves, and suggestive trends for diversity depending on small bowel length) offer insights into the effects of SBS and disease severity on GI community composition. While it is impossible to determine from this study whether altered microbial diversity is a by-product of SBS, or whether decreased microbial diversity might worsen the clinical consequences of SBS, our finding could be interpreted to suggest that efforts aimed at enhancing GI microbial diversity might offer some hope for ameliorating the clinical consequences of SBS. The analysis is limited and it is impossible to establish a causal relationship, but our findings that specific taxa were associated with both having a short bowel and with a worse state of health in patients with short bowels–a requirement for PN–raises interesting questions. We found that there were differences in specific taxa in different disease states, for example, in patients with short bowels compared to patients with bowels of normal length, and in patients that required or did not require PN. If a requirement for PN can be viewed as an indicator of disease severity, it might then be reasonable to hypothesize that providing or encouraging the growth of a microbe that was present a low levels in patients requiring PN or targeting a microbe that was present at high levels in patients requiring PN, for example with specific antibiotics or enteral antibodies, might have an effect on the clinical manifestations of SBS, and those kinds of hypotheses would be testable.

## Supporting information

S1 Table **Table A.** Differential abundance result table from the DESeq2 analysis comparing, for all patients, bacterial taxa in SS for patients on or off PN. The columns are as follows: column 1, OTU number; column 2 (*baseMean*), average of the normalized count values; column 3 (*log2FoldChange*), log2 fold change; column 4 (*lfcSE*), standard error; column 5 (*stat*), Wald statistic; column 6 (*pvalue*), Wald test p value; column 7 (*padj*), p-value adjusted for multiple testing using the Benjamini-Hochberg correction; columns 8 through 14, taxonomic classification at kingdom, phylum, class, order, family, genus and species levels; column 15 (*taxonomy*), taxonomic name; column 16 (OTUID), taxonomic name and OTU number; column 17 (*rep_seq*), OTU representative sequence.
**Table B.** Differential abundance result table from the DESeq2 analysis comparing, for SBS patients only, on vs. off PN. The columns are as follows: column 1, OTU number; column 2 (*baseMean*), average of the normalized count values; column 3 (*log2FoldChange*), log2 fold change; column 4 (*lfcSE*), standard error; column 5 (*stat*), Wald statistic; column 6 (*pvalue*), Wald test p value; column 7 (*padj*), p-value adjusted for multiple testing using the Benjamini-Hochberg correction; columns 8 through 14, taxonomic classification at kingdom, phylum, class, order, family, genus and species levels; column 15 (*taxonomy*), taxonomic name; column 16 (OTUID), taxonomic name and OTU number; column 17 (*rep_seq*), OTU representative sequence.
**Table C.** Differential abundance result table from the DESeq2 analysis comparing SBS off PN vs. normal patients. The columns are as follows: column 1, OTU number; column 2 (*baseMean*), average of the normalized count values; column 3 (*log2FoldChange*), log2 fold change; column 4 (*lfcSE*), standard error; column 5 (*stat*), Wald statistic; column 6 (*pvalue*), Wald test p value; column 7 (*padj*), p-value adjusted for multiple testing using the Benjamini-Hochberg correction; columns 8 through 14, taxonomic classification at kingdom, phylum, class, order, family, genus and species levels; column 15 (*taxonomy*), taxonomic name; column 16 (OTUID), taxonomic name and OTU number; column 17 (*rep_seq*), OTU representative sequence.
**Table D:** Differential abundance result table from the DESeq2 analysis comparing normal patients vs. SBS patients not receiving PN for JA samples. The columns are as follows: column 1, OTU number; column 2 (*baseMean*), average of the normalized count values; column 3 (*log2FoldChange*), log2 fold change; column 4 (*lfcSE*), standard error; column 5 (*stat*), Wald statistic; column 6 (*pvalue*), Wald test p value; column 7 (*padj*), p-value adjusted for multiple testing using the Benjamini-Hochberg correction; columns 8 through 14, taxonomic classification at kingdom, phylum, class, order, family, genus and species levels; column 15 (*taxonomy*), taxonomic name; column 16 (OTUID), taxonomic name and OTU number; column 17 (*rep_seq*), OTU representative sequence.
**Table E:** Differential abundance result table from the DESeq2 analysis comparing normal patients vs. SBS patients not receiving PN for SS samples. The columns are as follows: column 1, OTU number; column 2 (*baseMean*), average of the normalized count values; column 3 (*log2FoldChange*), log2 fold change; column 4 (*lfcSE*), standard error; column 5 (*stat*), Wald statistic; column 6 (*pvalue*), Wald test p value; column 7 (*padj*), p-value adjusted for multiple testing using the Benjamini-Hochberg correction; columns 8 through 14, taxonomic classification at kingdom, phylum, class, order, family, genus and species levels; column 15 (*taxonomy*), taxonomic name; column 16 (OTUID), taxonomic name and OTU number; column 17 (*rep_seq*), OTU representative sequence.
(XLSX)
